# Feline mammary carcinoma stem cells are tumorigenic, radioresistant, chemoresistant and defective in activation of the ATM/p53 DNA damage pathway

**DOI:** 10.1016/j.tvjl.2012.10.021

**Published:** 2013-06

**Authors:** L.Y. Pang, T.M. Blacking, R.W. Else, A. Sherman, H.M. Sang, B.A. Whitelaw, T.R. Hupp, D.J. Argyle

**Affiliations:** a The Roslin Institute and Royal (Dick) School of Veterinary Studies, University of Edinburgh, Easter Bush, Edinburgh EH25 9RG, UK; b Cancer Research UK Cell Signalling Unit, Institute of Genetics and Molecular Medicine, University of Edinburgh, Edinburgh EH4 2XR, UK

**Keywords:** Feline mammary carcinoma, Cancer stem cell, p53, Epithelial–mesenchymal transition

## Abstract

Cancer stem cells were identified in a feline mammary carcinoma cell line by demonstrating expression of CD133 and utilising the tumour sphere assay. A population of cells was identified that had an invasive, mesenchymal phenotype, expressed markers of pluripotency and enhanced tumour formation in the NOD-SCID mouse and chick embryo models. This population of feline mammary carcinoma stem cells was resistant to chemotherapy and radiation, possibly due to aberrant activation of the ATM/p53 DNA damage pathway. Epithelial–mesenchymal transition was a feature of the invasive phenotype. These data demonstrate that cancer stem cells are a feature of mammary cancer in cats.

## Introduction

Mammary tumours frequently develop in female domestic cats and are the third most commonly reported cancer in this species (Hayes and Mooney, 1985). Approximately 80% of these tumours are malignant, rapidly metastasising and often fatal. Surgery is the most widely used treatment (Vail and MacEwen, 2000). The mean age of development of mammary neoplasia in cats is 10–12 years (Zappulli et al., 2005). Cats that are ovariohysterectomised <6 months of age have a reduced risk of developing mammary tumours when compared to intact females, indicating that hormones are involved in the pathogenesis of the disease (Overley et al., 2005). Collectively, this evidence suggests that feline mammary carcinoma is similar to human breast cancer in terms of relative age of onset, incidence, histopathology, biological behaviour and patterns of metastasis.

Accumulating evidence supports the cancer stem cell (CSC) model of tumorigenesis, whereby a neoplasm can be viewed as the result of aberrant organogenesis driven by a subpopulation of CSCs, defined by capacity for self-renewal, differentiation potential and tumour-initiating ability. It has been proposed that CSCs are responsible for driving tumour growth and for recurrence after conventional therapeutic modalities (Pang and Argyle, 2009). Evidence for the existence of CSCs has been found in human acute myeloid leukaemias (Bonnet and Dick, 1997), breast cancers (Al-Hajj et al., 2003) and glioblastomas (Singh et al., 2003). In companion animal medicine, CSCs have been identified in canine osteosarcomas (Wilson et al., 2008), mammary carcinomas (Pang et al., 2011), gliomas (Stoica et al., 2009) and feline squamous cell carcinomas (Pang et al., 2012).

Stem-like cells have been isolated from feline mammary carcinoma (FMC) tissue and characterised in terms of sphere and tumour forming ability (Barbieri et al., 2012). In the present study, we used a FMC cell line to characterise feline mammary CSCs in terms of increased expression of specific stem cell markers, higher potential for mammosphere formation, invasiveness and resistance to radiation and chemotherapy compared to the bulk tumour population.

## Materials and methods

### Cell culture and mammosphere formation

FMC cells (Norval et al., 1985) were cultured in Dulbecco’s modified Eagle’s medium (DMEM; Invitrogen) supplemented with 10% fetal bovine serum (FBS) and 100 μg/mL streptomycin (Gibco). To assess anchorage independent culture, FMC cells were plated as single cells and grown as described previously (Pang et al., 2012).

### RNA extraction and reverse transcription PCR

Total cellular RNA was extracted using the RNeasy kit (Qiagen) and RNA quality was determined by measurement of absorbance at 260 nm (A_260_). Semi-quantitative reverse transcriptase (RT)-PCR analysis of mRNA expression of *Oct4*, *Nanog* and *STAT3* was performed as described previously (Pang et al., 2011). Semi-quantitative analysis was carried out using ImageJ.^1^

### Western blot analysis

Cells were lysed in in 7 M urea, 0.1 M dithiothreitol (DTT), 0.05% Triton X-100, 25 mM NaCl and 20 mM 4-(2-hydroxyethyl)-1-piperazine ethane sulphonic acid (HEPES; pH 7.5). Equal amounts of protein were separated by sodium dodecyl sulphate–polyacrylamide gel electrophoresis (SDS PAGE), transferred to Hybond-C nitrocellulose membrane (Amersham Pharmacia Biotech) and hybridised to an appropriate primary antibody (Table 1), followed by horseradish peroxidase (HRP)-conjugated secondary antibodies: rabbit anti-mouse immunoglobulin G (IgG), rabbit anti-goat IgG and porcine anti-rabbit IgG (DakoCytomation; all at 1:1000 dilution). Immunoreactivity was detected by chemiluminescence.

### Cell sorting

The Aldefluor Test Kit (Aldagen Stem Cell Technologies) was used to determine aldehyde dehydrogenase (ALDH) activity. Diethylaminobenzaldehyde (DEAB), a substrate for class 3 ALDH enzymes and hence a competitive inhibitor of ALDH1, was used as a negative control.

Flow cytometry with a fluorescence activated cell sorter (FACS Calibur; BD Biosciences) was used to identify a side-population phenotype (SP) of FMC cells able to efflux Hoeschst 33342 dye by ABC transporter activity. FMC cells were resuspended at 1 × 10^6^ cells/mL in pre-warmed DMEM with 5 μg/mL Hoechst 33342 (Sigma–Aldrich), with or without 50 μM verapamil (Sigma–Aldrich), and incubated at 37 °C for 90 min. Cells were washed with ice-cold Hank’s balanced salt solution (HBSS) and treated with 2 μg/mL propidium iodide (Invitrogen), which was used to gate viable cells. After excitation at 350 nm, fluorescence was measured at 424/440 nm and 675 nm for detection of blue and red, respectively.

A CD133 cell isolation kit (Miltenyi Biotec) was used for magnetic cell sorting (MACS).

### Invasion assay

The cell invasion ability of isolated cells was determined using a collagen-based cell invasion assay kit (QCM, Millipore). Cells were seeded into the upper inserts at 1 × 10^5^ cells per insert in serum-free DMEM and incubated at 37 °C in 5% CO_2_ for 48 h. Non-invading cells were removed. Cells that migrated through the gel insert to the lower surface were stained and quantified by colorimetric measurement at 560 nm.

### In vitro assays for tumorigenicity

The sphere forming ability of sorted CD133^−^ and CD133^+^ cells was determined as described by Pang et al. (2012). The in vitro proliferation rate of isolated CD133^−^ and CD133^+^ cells was determined by seeding cells in 60 mm dishes at 4 × 10^4^ cells per dish in triplicate. Cell proliferation was monitored 24 and 48 h after plating. Live cells were quantified by staining with trypan blue (Invitrogen). Cell viability assays were performed using CellTiterGlo (Promega). Both assays were normalised to the number of cells initially seeded.

### In vivo assays for tumorigenicity

Non-obese diabetic (NOD)-severe combined immunodeficient (SCID) mice were purchased from Charles River Laboratories. In vivo experiments were approved by the Home Office, UK (licence number PPL 60/3553, valid until 16 June 2011). Single cell suspensions of viable FMC cells (1 × 10^6^: *n* = 6; 1 × 10^4^: *n* = 6) were prepared in a 100 μL 1:1 mixture of medium (DMEM supplemented with 10% FBS and 100 μg/mL streptomycin):matrigel (Sigma) and inoculated subcutaneously into the right flank of each mouse. Tumour formation was monitored from 1 week after inoculation. After 3 weeks, all mice were euthanased and tumour volume was assessed using a digital caliper to measure height, width and depth. Tumours were fixed in 10% neutral buffered formalin, then processed routinely for histopathology.

### Chick embryo chorioallantoic membrane assay

To examine the in vivo migration potential of FMC stem cells, we utilised the chick embryo chorioallantoic membrane (CAM) model (Kunzi-Rapp et al., 2001). Chick embryos were inoculated with fluorescently labelled dissociated mammospheres or parental adherent cells directly onto their CAM at day 7 of development. Fertilised Isa Brown layer strain chicken eggs were obtained from the Roslin Institute, Easter Bush, Edinburgh, UK. Embryos from freshly laid eggs were transferred into large surrogate chicken eggshells (Perry, 1988), sealed and incubated at 37.5 °C in 60% relative humidity with turning through 30° for 7 days. Single cell suspensions of adherent cells and mammospheres were labelled with PKH26, a red fluorescent live cell membrane dye (Sigma–Aldrich); 2 × 10^7^ single cells were washed in serum-free media and then incubated with 4 μM PKH26 dye for 5 min at room temperature. The reaction was stopped by adding an equal volume of serum. Labelled cells were seeded in complete media, then single cell suspensions of viable cells (1 × 10^5^: *n* = 4) were prepared in 25 μL of a 1:1 mixture of serum-free medium (DMEM):matrigel (Sigma) and inoculated directly onto the CAM. The embryos were resealed and incubated without turning. Tumour growth and location were determined at 10 days by microscopic evaluation.

### Radiation and cytotoxic drug sensitivity

Mammospheres were disaggregated into single cells prior to irradiation in culture medium (DMEM supplemented with 10% FBS and 100 μg/mL streptomycin) using a Faxitron cabinet X-ray system 43855D at a central dose rate of 2 Gy/min. Cells were treated with 1 nM doxorubicin (Adriamycin, Pharmacia/Pfizer; catalogue number 16151), mitoxantrone hydrochloride (Novantrone, Baxter Healthcare; catalogue number 2636B2049) or vincristine sulfate (Oncovin, Hospira; catalogue number 61703-309-06). All drugs were of pharmaceutical grade and suitable for injection. All drugs were diluted in medium (DMEM supplemented with 10% FBS and 100 μg/mL streptomycin) immediately before use. Cytotoxicity was measured using the CellTiter-Glo Luminescent Cell Viability Assay (Promega), as described by Pang et al. (2012). Data were averaged and normalised against the average signal of untreated/vehicle control treated samples.

FMC cells and mammospheres were trypsinised into single cells and seeded at 500 cells/10 cm plate for colony formation assays. The cells were irradiated at 0, 1, 2.5 and 5 Gy in suspension. Plates were incubated at 37 °C in a humidified CO_2_ incubator until colonies were visible. Growth media was changed once per week. Colonies were fixed by incubating with ice cold methanol for 5 min at room temperature, then stained with Giemsa (Invitrogen).

### Subcellular proteome extraction

The Subcellular Proteome Fractionation Kit (Thermo Scientific) was used to extract proteins from mammalian cells according to their subcellular localisation. All fractions were stored at −70 °C and analysed by Western blot analysis (see above).

### Statistical analysis

Analysis of variance (ANOVA), Student’s *t* test and the Mann–Whitney *U* test were performed using Minitab. Data are expressed as means ± standard deviation (SD). The criterion for significance was *P* < 0.05.

## Results

### Isolation of cells with stem cell features from feline mammary carcinoma cells

**Fig. 1:**
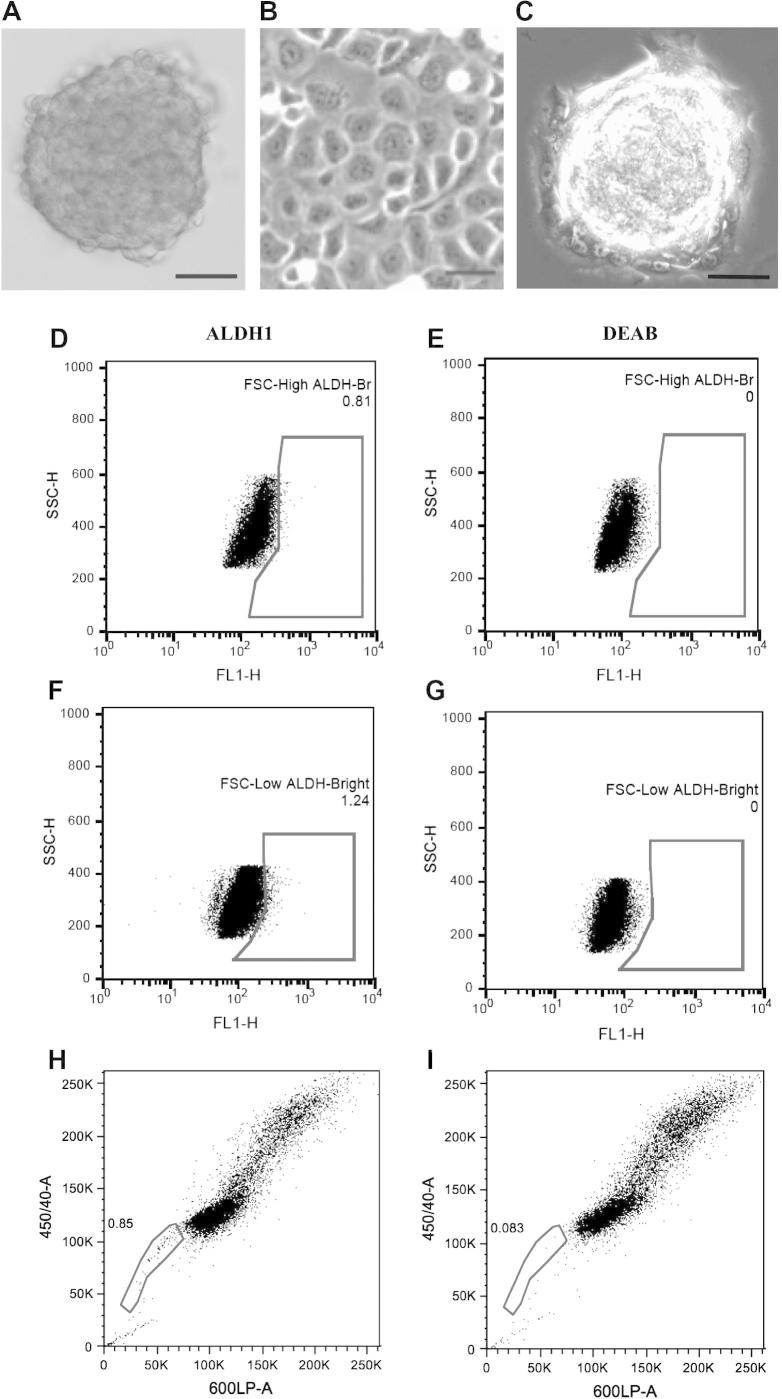
Isolation and identification of putative cancer stem cells showing that mammospheres can be derived from a feline mammary carcinoma cell line. (A) Spherical colonies derived from parental cells cultured without serum in an anchorage dependent manner. Scale bar = 50 μm. (B) Parental adherent cells. Scale bar = 20 μm. (C) Mammosphere colony removed from suspension culture and allowed to attach to a substratum. Adherent cells can be seen expanding from the sphere. Scale bar = 50 μm. Identification of aldehyde dehydrogenase 1^+^ (ALDH1^+^) and side-population cells from feline mammary carcinoma cells. The percentage of ALDH1^+^ cells isolated from FSH^High^ and FSH^Low^ populations was 0.81% (D) and 1.24% (F), respectively. Diethylaminobenzaldehyde (DEAB) was used as a negative control (E and G). A Hoechst 33342 dye exclusion assay on feline mammary carcinoma cells identified a side-population of 0.85% (H). Incubation with verapamil abolished the side population (I).

FMC cells formed mammospheres after 7–10 days in culture at low density (Fig. 1A). These cells were expanded in vitro and formed mammospheres for 20 subsequent passages. Adherent FMC cells had a cobblestone-like epithelial appearance (Fig. 1B). Mammospheres transferred back into adherent conditions attached and grew as monolayers (Fig. 1C).

ALDH activity of FMC cells was measured to assess the presence and size of a stem cell-like population. A small population of ALDH^+^ FMC cells was isolated (Fig. 1D–G). The SP fraction was 0.85% of total cells (Fig. 1H). Incubation in the presence of 100 μM verapamil, an ABC transporter inhibitor, abolished the SP (Fig. 1I), indicating that the FMC cell line contains a subpopulation of stem-like cells.

### Mammosphere forming ability of CD133^+^ feline mammary carcinoma cells

**Fig. 2:**
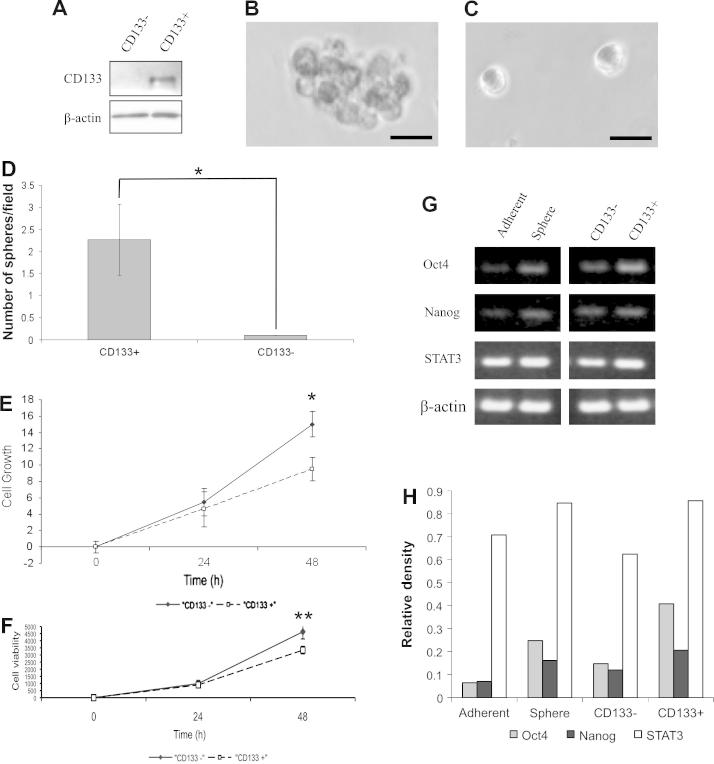
Characterisation of a subpopulation of CD133^+^ feline mammary carcinoma cells enriched for spheroid forming ability. A small population of CD133^+^ cells existing in feline mammary carcinoma cells were isolated by magnetic cell sorting. (A) CD133^+^ and CD133^−^ cell fractions were processed and analysed for the expression of CD133 (120 kDa) by Western blot analysis. Single cells sorted for CD133 expression were evaluated for the potential to form spherical colonies in serum-free medium. Spheres formed from CD133^+^ cells (B) but not CD133^−^ cells (C). Scale bars = 20 μM. (D) The numbers of the resultant spherical colonies from CD133^+^ and CD133^−^ cells were counted. Data are representative of three independent experiments (*P* = 0.01). CD133^−^ cells have a higher growth potential than CD133^+^ cells, as illustrated by (E) trypan blue exclusion assay (^*^P = 0.02) and (F) cell viability assay (^**^P = 0.018). (G) Reverse transcriptase (RT)-PCR analysis of *Oct4*, *Nanog*, *STAT3* and β-actin gene expression levels. (H) Quantification of RT-PCR results using ImageJ to determine the relative density of the bands compared to β-actin loading controls.

CD133^+^ cells (mean ± SD: 0.72 ± 0.25%; 10 replicates) were isolated from FMC cells by MACS. Western blot analysis confirmed that CD133 expression was restricted to the CD133^+^ cell population (Fig. 2A). CD133^+^ cells formed more and larger mammospheres (Fig. 2B) than CD133^−^ cells (Fig. 2C) when cultured in serum-free medium (*P* = 0.01). In addition, CD133^−^ cells gave rise to more cells after 48 h than CD133^+^ cells, as measured by trypan blue exclusion (*P* = 0.02, Fig. 2E) and cellular adenosine triphosphate (ATP) measured by cell viability assay (*P* = 0.018, Fig. 2F). These data indicate that CD133^+^ cells are more representative of stem cells than their CD133^−^ counterparts. Using semi-quantitative RT-PCR, mammospheres and CD133^+^ cells expressed higher mRNA levels of the embryonic stem cell (ESC)-specific genes *Oct4*, *Nanog* and *STAT3* compared to adherent cells and CD133^−^ cells, respectively (Fig. 2G and H).

### In vivo tumorigenic potential of mammospheres

**Fig. 3:**
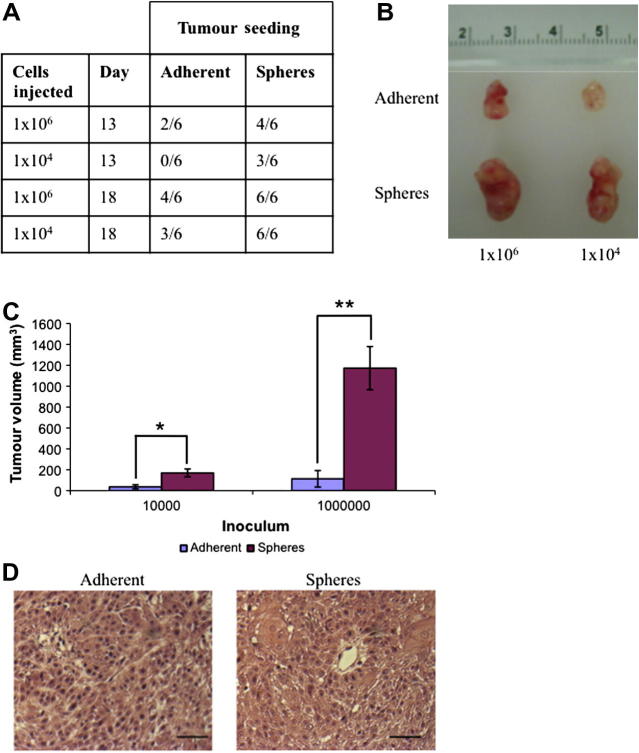
Mammospheres are enriched for high tumorigenicity in vivo. Dissociated mammospheres and adherent cells were injected SC into NOD-SCID mice. The number of mice with a tumour was tabulated (A) and representative images of tumours 3 weeks after inoculation are shown (B). (C) Tumour volumes generated by spheres and adherent cells were measured and recorded 3 weeks after inoculation: 10^4^: ^*^P < 0.04; 10^6^: ^**^P < 0.001. (D) Haematoxylin and eosin staining of histological sections of representative xenograft tumour sections. Scale bar = 50 μm.

Single cells were expanded to 1 × 10^6^ (*n* = 6) or 1 × 10^4^ (*n* = 6) cells and implanted by subcutaneous injection into the right flanks of female NOD-SCID mice. Thirteen days after inoculation, dissociated mammosphere cells initiated growth of more tumours (3/6) than parental adherent cells (0/6) with the implantation of 1 × 10^4^ (*n* = 6) cells (Fig. 3A). Three weeks after inoculation, tumours derived from dissociated mammospheres were significantly larger than those from parental adherent cells (10^4^ cells: 4.7-fold increased size, *P* = 0.03; 10^6^ cells: 10-fold increased size, *P* = 0.005; Fig. 3B and C). However, despite differences in size, both types of tumour were poorly differentiated, with no substantive qualitative differences (Fig. 3D).

### Invasiveness of mammospheres and CD133^+^ cells

**Supplementary Fig. 1:**
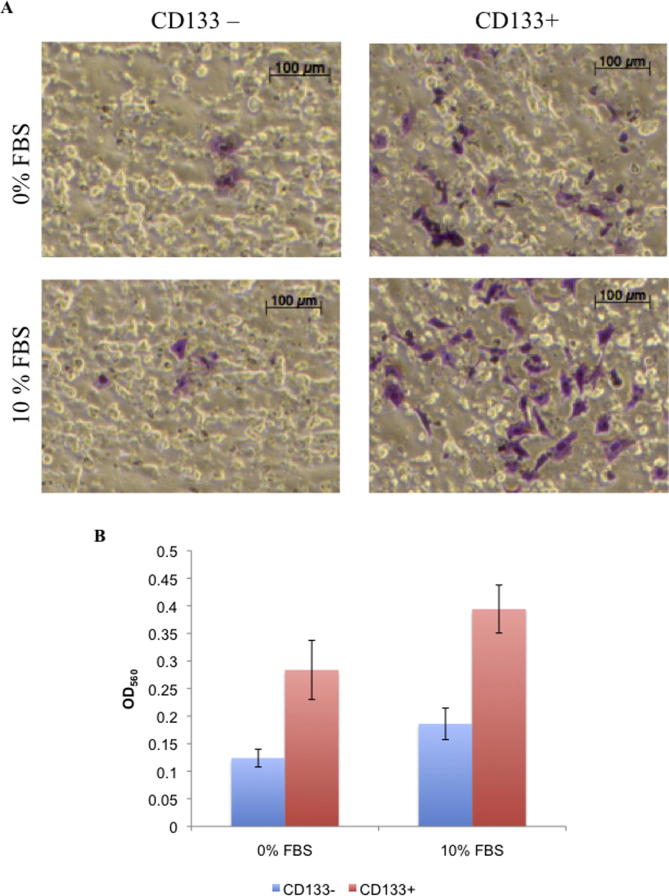
CD133^+^ cells show an increased in vitro invasive potential compared to CD133^−^ cells. (A) Examination of invasive ability of sorted CD133^+^ cells utilising a collagen-based cell invasion assay. Fetal bovine serum (FBS) was used as a chemoattractant. Scale bar = 100 μm. (B) Quantification of invading cells.

**Fig. 4:**
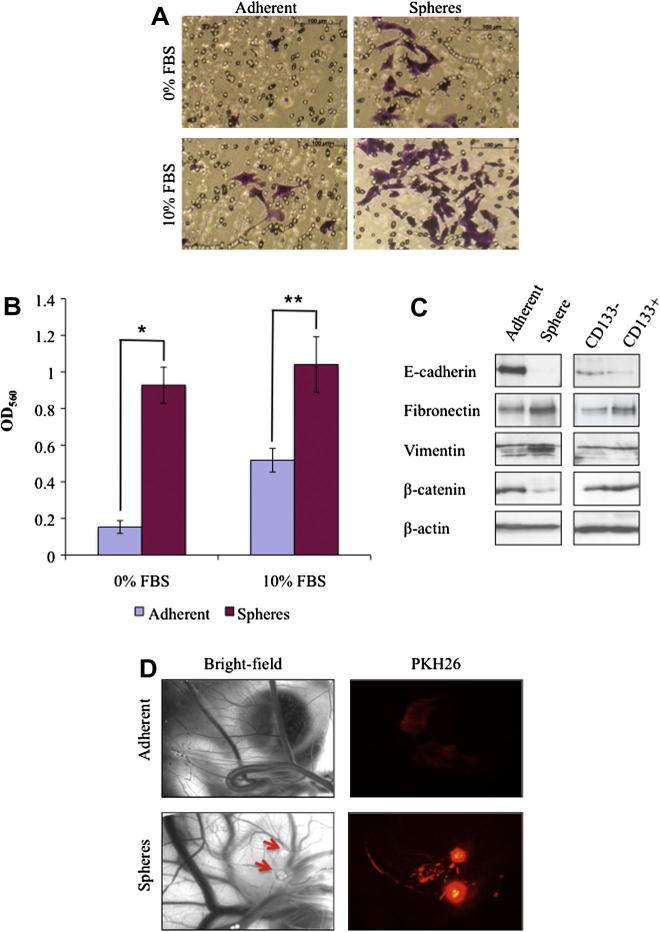
Putative cancer stem cells show an increased in vitro and in vivo invasive potential and exhibit mesenchymal phenotypes. (A) Examination of invasive ability of sorted CD133^+^ cells utilising a collagen-based cell invasion assay. FBS, fetal bovine serum. (B) Quantification of invading cells. ^*^P < 0.001; ^**^P < 0.005; OD_560_, Optical density at 560 nm. (C) Western blot analysis of E-cadherin, fibronectin, vimentin and β-catenin, with β-actin as a loading control. (D) Evaluation of in vivo tumorigenesis and invasiveness in a chick embryo model. Bright field image of the chorioallantoic membrane (CAM) 3 days after inoculation with feline mammary carcinoma cells (left panel) and fluorescence image of tumour cells 3 days after inoculation (right panel). Red arrows indicate opaque plaques representative of tumour growth.

Cells derived from mammospheres were significantly more invasive than parental adherent cells both with (*P* < 0.005) and without (*P* < 0.001) FBS (Fig. 4A and B). Similar results were obtained with matched CD133^+^ and CD133^−^ cells (see Appendix A: Supplementary Fig. 1).

Western blot analysis demonstrated that mammospheres and CD133^+^ cells exhibited a shift towards mesenchymal phenotypes, including down-regulation of the epithelial markers E-cadherin and β-catenin, and up-regulation of the mesenchymal markers fibronectin and vimentin (Fig. 4C).

Tumours formed in 100% of chick embryo CAMs inoculated with dissociated mammospheres, but not in CAMs inoculated with parental adherent cells (Fig. 4D). Using fluorescence microscopy, tumours derived from mammospheres were brightly fluorescent and had radiated out from the site of inoculation, invading the surrounding blood vessels of the CAM. In contrast, adherent cells were weakly fluorescent and were localised to the initial site of inoculation (Fig. 4D).

### In vitro resistance of mammospheres and CD133^+^ cells to irradiation and chemotherapeutic agents

**Fig. 5:**
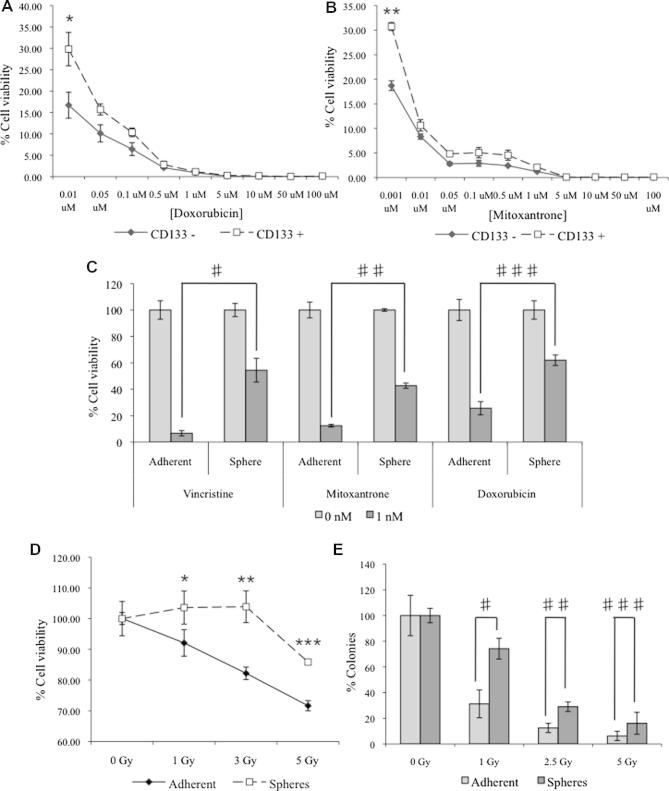
Mammospheres and CD133^+^ cells are resistant to conventional therapeutic agents. CD133^+^ and CD133^−^ cells were treated with increasing concentrations of (A) doxorubicin (^*^P = 0.026) or (B) mitoxantrone (^**^P = 0.029) and cell viability was assayed 48 h post-treatment. Mammospheres and adherent cells were treated with 1 nM vincristine (^#^*P* < 0.003), 1 nM mitoxantrone (^##^*P* < 0.001) or 1 nM doxorubicin (^###^*P* < 0.001) and cell viability relative to untreated controls (0 nM) was assayed 48 h after treatment (C). Mammospheres and adherent cells were treated with increasing doses of γ-radiation and assayed for (D) cell viability (^*^P = 0.029; ^**^P = 0.015; ^***^P = 0.001) and (E) colony formation ability (^#^*P* = 0.009; ^##^*P* = 0.003; ^###^*P* = 0.089).

In the cytotoxicity assay, CD133^+^ FMC cells were more resistant to doxorubicin (Fig. 5A; *P* = 0.026) and mitoxantrone (Fig. 5B; *P* = 0.029) than CD133^−^ cells. Similarly, mammospheres were more resistant than parental adherent cells to 1 nM doxorubicin (*P* < 0.003), 1 nM mitoxantrone (*P* < 0.001) and 1 nM vincristine (*P* < 0.001) (Fig. 5C).

Adherent parental cells exhibited a dose dependent decrease in viability with increasing doses of irradiation, whereas mammosphere viability was unaffected until a dose of 5 Gy was reached (Fig. 5D). This was confirmed by clonogenic analysis (Fig. 5E, *P* < 0.009).

### Activation of DNA damage pathways in feline mammary carcinoma cells

**Fig. 6:**
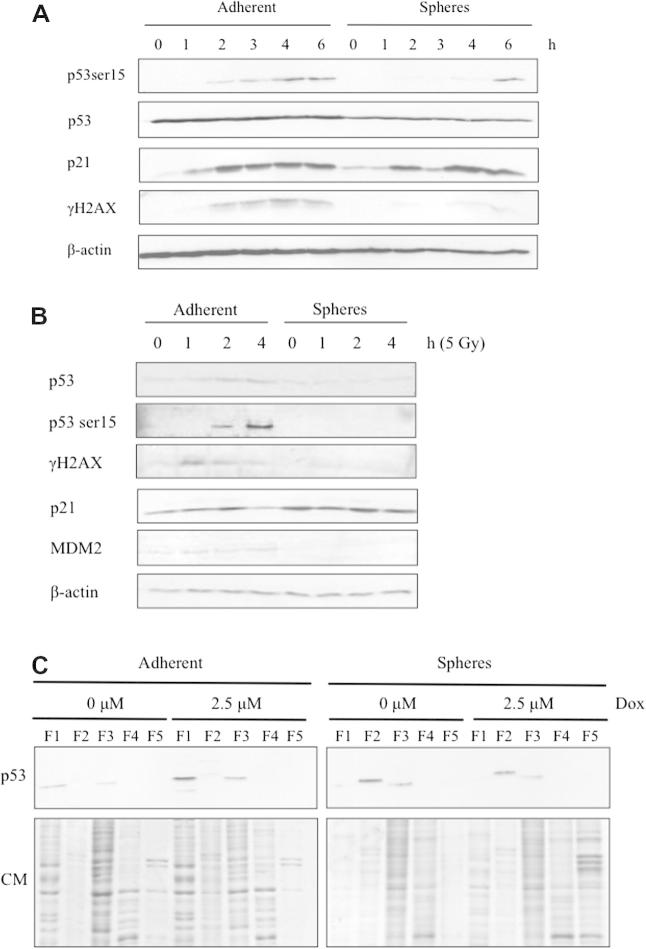
Feline cancer stem cells lack activation of the p53 DNA damage pathway in response to doxorubicin and ionising radiation. (A) Dissociated mammospheres and parental adherent cells were treated with 10 μM doxorubicin or dimethyl sulfoxide (DMSO), cells were harvested over the indicated time course and expression of p53 pathway related proteins was assessed. (B) Dissociated mammospheres and parental adherent cells were treated with 5 Gy ionising radiation, cells were harvested over the indicated time course and expression of proteins related to the p53 pathway was assessed. Dissociated mammospheres and adherent cells were incubated with 2.5 μM doxorubicin (Dox) or DMSO. Proteins were extracted according to their subcellular localisation: F1, cytosolic; F2, membranes/organelles; F3, nucleus; F4, nucleus; F5, cytoskeleton. Proteins from each fraction were resolved by SDS–PAGE and analysed by immunoblotting for p53; 30 μg was loaded per lane. Coomassie (CM) staining confirmed that protein expression profiles from each fraction were distinct; 5 μg was loaded per lane (C).

In response to doxorubicin, adherent cells showed a transient increase in phosphorylation of p53-serine15, an ATM target that is associated with activation of p53 transcriptional activity (Kastan et al., 1991), and of the p53 transcriptional target p21*^WAF1^*^/^*^Cip1^* at 2 h post-treatment (Fig. 6A).

Levels of γH2AX, an ATM target and a marker of DNA double strand breaks (Rogakou et al., 1998), similarly increased 2 h post-treatment in adherent cells (Fig. 6A), whereas in mammospheres treated with doxorubicin γH2AX could not be detected and phosphorylation of p53-serine15 was delayed until 6 h post-treatment. Similar results were obtained in response to irradiation (Fig. 6B).

In untreated parental adherent cells, p53 protein was detected at a low level and was associated with the cytosolic and nuclear fractions, whereas in untreated mammospheres p53 protein levels were relatively high and were predominantly associated with membranes and, to a lesser extent, the nucleus (Fig. 6C). Upon DNA damage, the p53 protein levels in adherent cells increased in both the cytoplasmic and nuclear fractions, whereas in mammospheres p53 protein levels and subcellular localisation remain unchanged (Fig. 6C).

## Discussion

The concept of CSCs is still evolving, but data implicating these cells in tumour maintenance and resistance to therapeutic agents suggest that there is value in targetting CSCs in combination with conventional therapies (Pang and Argyle, 2009). In this study, CSCs were isolated from a feline mammary carcinoma cell line by mammosphere formation. The ability of mammospheres to grow in anchorage-independent conditions and to form three-dimensional spherical structures in serum-free medium is recognised as a stem cell characteristic (Singh et al., 2003). The expression of ALDH1 by mammospheres correlates with the self-renewal properties of CSCs (Ginestier et al., 2007). We also isolated a CD133^+^ population of FMCs; previous studies have demonstrated that human CSCs can be enriched through the use of cell surface markers such as CD24, CD44 and CD133 (Singh et al., 2003; Bao et al., 2006a,b; Hermann et al., 2007; O’Brien et al., 2007).

Previously, we demonstrated that expression of CD44 in feline cancer cells is cell cycle dependent (Blacking et al., 2011) and therefore it is not appropriate to utilise CD44 as a CSC marker in this species. Furthermore, available antibodies against CD24 demonstrate inconsistent cross-reactivity in the feline system (data not shown). Putative tumour initiating cells previously have been shown to express the embryonic stem cell-specific genes *Oct4*, *Nanog* and *STAT3*, which are important for self-renewal, proliferation, and fate determining properties of stem cells (Wilson et al., 2008). In the present study, we showed that putative FMC CSCs, represented by CD133^+^ cells, express higher levels of ESC-specific genes. *STAT3* is persistently activated in a range of tumours and it is not surprising that STAT3 expression is also detectable in adherent and CD133*^−^* cells. Significantly, in xenograft mouse models we demonstrated that dissociated mammospheres can initiate tumour development in vivo better than parental adherent cells.

CSCs also may be responsible for mediating tumour metastasis. Recent evidence indicates that the epithelial–mesenchymal transition (EMT) pathway drives tumour invasion. EMT is a morphogenetic process in which cells lose their epithelial characteristics and gain mesenchymal properties (Cervantes-Arias et al., 2012). In the present study, we showed that CSCs shift towards a mesenchymal phenotype in comparison with parental cells. Using the chick embryo model, we also showed that feline CSCs are capable of invading the capillary system of the CAM. Further studies will be needed to determine whether these cells are capable of initiating and maintaining tumour growth at a distant site.

A proposed driver of tumour metastasis is the EMT pathway. Recent evidence indicates that in vitro induction of EMT in immortalised human mammary epithelial cells is sufficient to generate mesenchymal cells with properties of stem cells (Mani et al., 2008). Therefore, the EMT pathway may be sufficient both to enable invasion and to endow cancer cells with stem cell properties, which permits tumour establishment at a distant site. In our feline model, we showed that CSCs are inherently more invasive than parental cells, which is indicative of a mesenchymal phenotype. These data substantiate the role of EMT in invasiveness and metastatic dissemination; the ability to isolate metastatic CSCs from cell lines should facilitate studies of the molecular mechanisms by which CSCs mediate tumour metastasis.

An emerging characteristic of CSCs is their resistance to conventional therapeutic agents. The molecular mechanisms underlying the intrinsic resistance of CSCs to conventional therapies are still undefined. In the feline model system, we investigated the ATM-p53 DNA damage pathway. Human and mouse ESCs lack the cell cycle G1/S checkpoint and fail to undergo cellular senescence after DNA damage, which is effective in somatic cells (Aladjem et al., 1998). Classically, p53 protects the genome from accumulating genetic mutations by enabling faithful repair of DNA damage or by elimination of cells with excessive damage (Hupp et al., 2000). However, p53 is emerging as a major player in stem cell biology and has been implicated in suppression of pluripotency and cellular differentiation via suppression of Nanog (Zhao and Xu, 2010).

In the present study, we compared the p53 response of feline mammospheres to adherent cells in response to doxorubicin. Adherent cells showed a rapid response to doxorubicin treatment, whereas in mammospheres there was a delay in activation of p53. Our data indicate that the mechanism of ATM/p53 pathway inactivation is potentially due to mis-localisation of p53. We propose that, in the adherent cells, the rapid response of the p53 pathway is primarily to engage the G1/S checkpoint, whereas in mammospheres the aberrant kinetics of p53 activation and lack of phosphorylation of H2AX may indicate an alternative p53 response, such as induction of differentiation into other cell types that can undergo efficient p53-dependent cell cycle arrest or apoptosis.

We also observed a higher basal level of p21*^WAF1^*^/^*^Cip1^* in mammospheres compared to adherent cells. In response to DNA damage, p21*^WAF1^*^/^*^Cip1^* mediates G1 arrest (El-Deiry et al., 1994). However, p21*^WAF1^*^/^*^Cip1^* has a dual role in stem cell regulation, i.e. to limit self-renewal and to inhibit differentiation (Topley et al., 1999; Cheng et al., 2000). In this context, our data may indicate that the high basal level of p21*^WAF1^*^/^*^Cip1^* observed in the putative CSC population may inhibit differentiation and maintain the size and self-renewal potential of the CSC pool. The implications of classical DNA damage response proteins having additional roles in stem cell biology warrant further investigation.

## Conclusions

In this study, a FMC cell line was used to characterise feline mammary CSCs through expression of specific stem cell markers, higher potential for mammosphere formation, invasiveness and resistance to radiation and chemotherapy compared to the bulk tumour population. Similar to human breast cancer models, we were able to isolate a small population of cells with stem cell properties from a FMC cell line. Feline CSCs are capable of initiating tumour growth in the NOD-SCID mouse and chick embryo models. Feline CSCs are more invasive than other FMC cell line populations and display attributes of a mesenchymal cell state, which may enhance invasive capacity. These cells are also chemoresistant and radioresistant.

## Conflict of interest statement

None of the authors has any financial or personal relationships that could inappropriately influence or bias the content of the paper.

## Figures and Tables

**Table 1:** Primary antibodies used for immunolabelling.

Target	kDa	Species	Clonality	Supplier	Dilution
β-Actin	42	Mouse	Monoclonal	Abcam	1:2000
β-Catenin	94	Rabbit	Polyclonal	Abcam	1:1000
CD133 (k-18)	120	Goat	Polyclonal	Santa Cruz	1:500
E-cadherin	120	Mouse	Monoclonal	BD	1:1000
Fibronectin	240	Mouse	Monoclonal	BD	1:1000
γ-H2AX	16	Mouse	Monoclonal	Abcam	1:1000
MDM2 (4B2)	90	Mouse	Monoclonal	Moravian Biotechnology	1:1000
p21 (Ab-1)	21	Mouse	Monoclonal	Calbiochem	1:1000
p53 (DO12)	53	Mouse	Monoclonal	Moravian Biotechnology	1:1000
Phospho-p53ser15	53	Mouse	Monoclonal	Cell Signaling	1:1000
Twist	28	Rabbit	Polyclonal	Santa Cruz	1:500
Vimentin	57	Mouse	Monoclonal	Abcam	1:500

## Footnotes

1 See: http://rsbweb.nih.gov/ij/.
