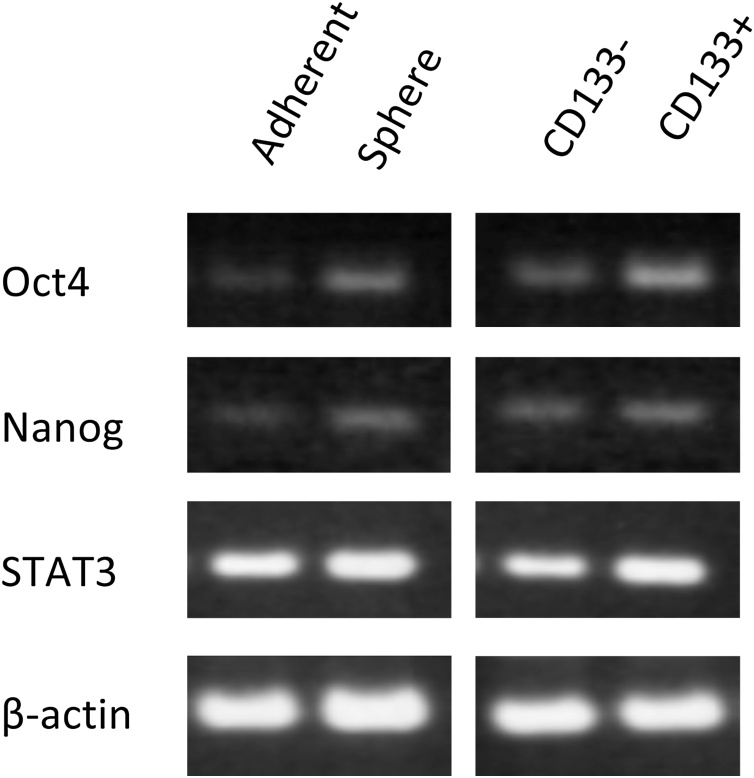# Corrigendum to “Feline mammary carcinoma stem cells are tumorigenic, radioresistant, chemoresistant and defective in activation of the ATM/p53 DNA damage pathway” [The Veterinary Journal 196 (2013) 414–423]

**DOI:** 10.1016/j.tvjl.2021.105744

**Published:** 2021-10

**Authors:** L.Y. Pang, T.M. Blacking, R.W. Else, A. Sherman, H.M. Sang, B.A. Whitelaw, T.R. Hupp, D.J. Argyle

**Affiliations:** a The Roslin Institute and Royal (Dick) School of Veterinary Studies, University of Edinburgh, Easter Bush, Edinburgh EH25 9RG, UK; b Cancer Research UK Cell Signalling Unit, Institute of Genetics and Molecular Medicine, University of Edinburgh, Edinburgh EH4 2XR, UK

The authors regret that in the original article there was a mistake in Figure 2G where the beta-actin band was repeated and horizontally flipped. The authors apologise for this error and state that this does not change the scientific conclusions of this article in anyway.

Please see below for the correct Figure 2G:

**Figure:**